# Analysis of Caudal Regression Syndrome: A Case Report From Bulgaria

**DOI:** 10.7759/cureus.69985

**Published:** 2024-09-23

**Authors:** Elitsa Gyokova, Eleonora Hristova-Atanasova, Merve Yesilyer

**Affiliations:** 1 Obstetrics and Gynaecology, University Hospital Saint Marina - Pleven, Pleven, BGR; 2 Obstetrics and Gynaecology, Medical University of Pleven, Pleven, BGR; 3 Social Medicine and Public Health, Medical University of Plovdiv, Plovdiv, BGR; 4 Faculty of Medicine, Medical University of Pleven, Pleven, BGR

**Keywords:** congenital abnormalities, diabetes mellitus management, prenatal diagnosis, rare diseases, regression caudal syndrome

## Abstract

A rare congenital condition known as caudal regression syndrome (CRS) or caudal dysplasia sequence (CDS) is defined by deformity of the caudal (lower) half of the body, which can have different effects on skeletal, neurological, gastrointestinal, and genitourinary systems. A 19-year-old G1P0 woman presented for a fetal anomaly scan at 27+6 weeks of gestation due to suspected oligohydramnios. The patient reported a history of maternal diabetes type 1 on insulin for the past 10 years. She presented with severe generalized edema and hypertension that was not reported till the first appointment with us with a blood pressure of 160/90 mmHg. Despite the current situation, the patient was also a severe smoker during pregnancy, with up to 15 cigarettes per day. In her recent blood glucose level diary, she noted poor diabetes control, with glucose levels in the range of 22 to 26 mmol/L. In the following report, we demonstrate that prenatal ultrasonography can detect this rare but important anomaly. Additionally, this case study highlights the significance of conducting a thorough ultrasonographic evaluation in mid-gestation to effectively manage pregnancies impacted by insulin-dependent diabetes mellitus.

## Introduction

A rare congenital condition known as caudal regression syndrome (CRS) or caudal dysplasia sequence (CDS) is defined by deformity of the caudal (lower) half of the body, which can have different effects on skeletal, neurological, gastrointestinal, and genitourinary systems [1,2].

Depending on the severity of the malformation, clinical findings can range from asymptomatic and lacking inferior coccygeal segments to a complete absence of the coccygeal, sacral, lumbar, and even inferior thoracic vertebrae. The related orthopedic anomalies can include pelvic deformity, kyphoscoliosis, agenesis of one or more ribs, dislocation of the hips, flexion contractures of the knees and hips, and deformities of the feet. Anorectal atresia, inguinal hernia, abdominal wall defects, gut malrotation, and imperforate anus are the most common gastrointestinal anomalies in CRS. This condition has also been associated with tracheoesophageal, rectovaginal, and recto-urethral fistulas. There is a possibility of vesicoureteral reflux, hydronephrosis, fused kidneys, renal agenesis, ectopic ureters, and transposition of external genitalia due to Mullerian duct agenesis. Besides all the mentioned conditions, neural tube defects, congenital heart defects, strabismus, and midline facial clefts have also been reported [2,3]. These severe defects may eventually lead to stillbirth or spontaneous abortion.

The exact etiology of CRS is still unknown. Despite the lack of a precise cause, researchers believe vascular hypoperfusion, genetic susceptibility, and maternal diabetes to be potential risk factors [4,5].

The following report demonstrates that prenatal ultrasonography can detect this rare but important anomaly. Additionally, this case study highlights the significance of conducting a thorough ultrasonographic evaluation mid-gestation to effectively manage pregnancies impacted by insulin-dependent diabetes mellitus.

## Case presentation

A fetal anomaly scan was performed on a 19-year-old G1P0 lady who was suspected of having oligohydramnios at 27+6 weeks of gestation. The patient presented to the University Hospital Saint Marina in Pleven, Bulgaria, in August 2023. The woman disclosed a history of insulin-treated type 1 maternal diabetes spanning the previous 10 years. She arrived with a blood pressure reading of 160/90 mmHg, significant generalized edema, and hypertension that was not disclosed until her initial visit with us. Despite the present circumstances, the patient smoked up to 15 cigarettes a day during her pregnancy. She reported inadequate diabetes management in her most recent blood glucose level diary, with glucose readings between 22 and 26 mmol/L.

**Figure 1:**
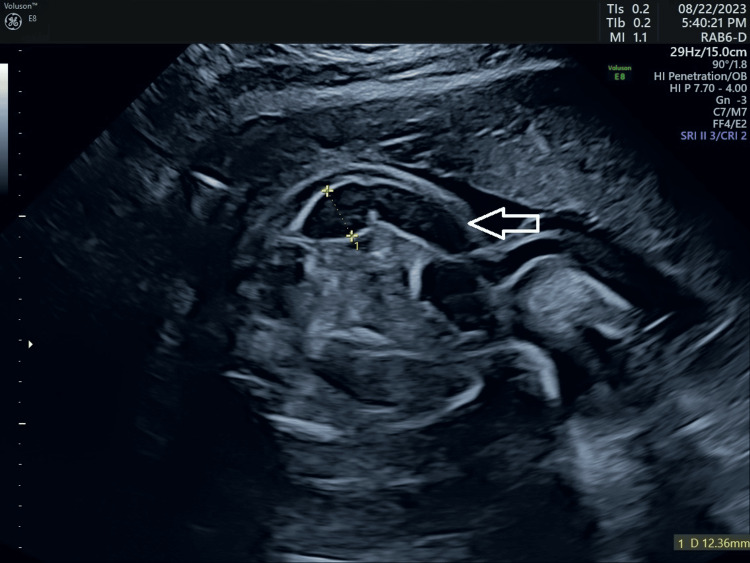
Enlarged intestinal loops with hyper echogenic bowels and suspected anorectal atresia at 27+6 gestational weeks

**Figure 2:**
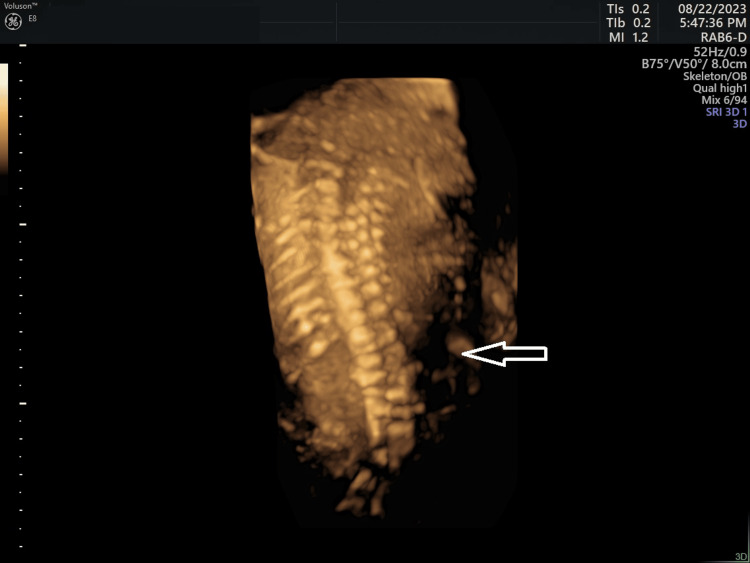
Right-sided kyphoscoliosis in the lumbar part at 27+6 gestational weeks

We performed a detailed ultrasound to evaluate the fetal anatomy. The scan was limited due to significant skin and tissue edema, oligohydramnios, and advanced pregnancy, resulting in poor image quality. The scan revealed several abnormalities, including right-sided kyphoscoliosis in the lumbar region, significantly enlarged intestinal loops with hyperechogenic bowels and suspected anorectal atresia, bilateral adducted thumbs, and right-sided clubfoot, along with possible joint arthrogryposis and a single umbilical artery (Figure 1, Figure 2).

The described findings and the data on poorly controlled diabetes cast doubt on the possibility of a caudal regression sequence diagnosis. A medically indicated termination of pregnancy by performing feticide was discussed due to the poor prognosis for this fetus, given the expected greatly reduced quality of life, and/or high risk of neonatal death, along with the mother's impaired general condition and the possibility of her condition deteriorating. The couple deliberated about the consequences of their choice but could not give their informed agreement.

The mother's health continued to deteriorate in the interim: she developed preeclampsia with blood pressure measurements reaching 170/100 mmHg. At 29 weeks, she had a premature rupture of the membranes and gave birth to a baby girl via emergency cesarean section because of a breech presentation. The premie was born at only 1170 grams with APGAR (appearance, pulse, grimace, activity, and respiration) scores of 3, 4, and 5 and multiple abnormalities, some of them as described previously: external ear aural atresia, adducted thumbs bilaterally, volvulus of small intestines and microcolon, right-sided kyphoscoliosis in the lumbar part between L1 and L5, clubfoot, and syndactyly on the lower extremities.

A multidisciplinary team of an endocrinologist, cardiologist, obstetrician, and psychologist provided treatment for the mother while she remained in the hospital. The hospital released her with a home therapy plan and a stable medical status 10 days after the birth. Regrettably, the hospital kept the baby longer due to numerous anomalies, the need for surgery, and its low birth weight.

## Discussion

There is no known cure for CRS, which is a complex, lifelong condition. Since it is a multidisciplinary syndrome, therapy must be comprehensive and complex, requiring a variety of professionals [2].

In the general population, the estimated prevalence is 1-3 newborn infants per 100,000 live births [1,2]. Babies born to diabetic mothers have a significantly higher prevalence rate, with an estimated 2 in 1000 newborns. The most distinctive congenital defect associated with maternal diabetes mellitus is believed to manifest in the first seven weeks of pregnancy [6]. It is uncertain what environmental variables contribute to the development of caudal regression syndrome, while a variety of plausible reasons have been proposed, such as alcohol, retinoic acid, hypoxia, and imbalances in amino acids. 

Abnormalities in cellular differentiation throughout the embryonic development stage could result in various spinal deformities. The caudal mesoderm experiences a developmental arrest during the embryonic phase, just before the fourth week of gestation, and this failure can lead to CRS [2,7]. As a result, to lower CRS rates, it is imperative to manage maternal diabetes and environmental problems before the time of fetal spinal development.

In their study, Mills et al. found that pregnancies evaluated before the crucial organogenesis period had a lower malformation rate than those evaluated later in pregnancy [8]. The study authors suggested seeking excellent glucose control before planning pregnancy or early in gestation, before organogenesis, as this may be therapeutic and will decrease the risk of developing CDS. Prenatal ultrasonography is the most common diagnostic method. In the presence of normal amniotic fluid, the diagnosis of sacral agenesis, a less severe form of caudal regression syndrome, can be confirmed by the absence of the lumbar spine and malformed lower extremities. However, oligohydramnios caused by renal agenesis can hinder the evaluation through ultrasound, whereas there might be enough amniotic fluid in the early stages of the second trimester to enable diagnosis. Therefore, ultrasonographic diagnosis during the early antenatal period is significant and enables less traumatic pregnancy termination.

Furthermore, MRI can be employed as an additional investigative technique when secondary syndrome findings (like oligohydramnios) obscure basic findings (like clubfoot) by impeding ultrasonography examination [7,9].

Given the uniqueness of each instance, it should be assessed separately, and an individual plan of management should be considered. The prognosis for children with caudal regression syndrome is determined by the degree of the lesion and the existence of any related abnormalities. Typically, infants, who survive, would have normal cognitive abilities but need significant medical support for urological and orthopedic issues [7].

The treatment aims to improve functionality and obtain the highest possible quality of life. The severity of the syndrome will be the criteria for the management plan. The majority of the anomalies that have been reported are surgically fixable or corrected. However, when combined, they greatly reduce the standard of living. As a result, to promptly arrange the best course of action, it is critical to establish an accurate diagnosis as soon as feasible [2].

## Conclusions

Maternal hyperglycemia and CRS are known to be related. Consequently, women with pregestational diabetes should manage their diabetes before becoming pregnant, and pregnant women with diabetes should undergo routine prenatal screening to prevent serious problems. When contemplating a pregnancy, comprehensive long-term therapy should begin as soon as feasible. The ideal scenario would be a multidisciplinary medical team offering sophisticated therapy to lower the fetal abnormalities related to maternal diabetes.
